# Achieved Gain and Subjective Outcomes for a Wide-Bandwidth Contact Hearing Aid Fitted Using CAM2

**DOI:** 10.1097/AUD.0000000000000661

**Published:** 2019-04-26

**Authors:** Tanya L. Arbogast, Brian C. J. Moore, Sunil Puria, Drew Dundas, Judith Brimacombe, Brent Edwards, Suzanne Carr Levy

**Affiliations:** 1 Earlens Corporation, Menlo Park, California, USA; 2 Department of Experimental Psychology, University of Cambridge, Cambridge, United Kingdom; 3 Department of Otology & Laryngology, Harvard Medical School, Boston, Massachusetts, USA; 4 Eaton-Peabody Laboratory of Auditory Physiology, Massachusetts Eye & Ear Infirmary, Boston, Massachusetts, USA; 5 National Acoustic Laboratories, Sydney, Australia.

**Keywords:** Auditory perception device, Contact hearing aid, Contact hearing device, Extended bandwidth hearing, Hearing aid, Hearing impaired

## Abstract

**Objectives:**

The objective of this study was to test the ability to achieve, maintain, and subjectively benefit from extended high-frequency amplification in a real-world use scenario, with a device that restores audibility for frequencies up to 10 kHz.

**Design:**

A total of 78 participants (149 ears) with mild to moderately-severe sensorineural hearing loss completed one of two studies conducted across eight clinical sites. Participants were fitted with a light-driven contact hearing aid (the Earlens system) that directly drives the tympanic membrane, allowing extended high-frequency output and amplification with minimal acoustic feedback. Cambridge Method for Loudness Equalization 2 - High Frequency (CAM2)-prescribed gains for experienced users were used for initial fitting, and adjustments were made when required according to participant preferences for loudness and comfort or when measures of functional gain (FG) indicated that more or less gain was needed. Participants wore the devices for an extended period. Prescribed versus adjusted output and gain, frequency-specific FG, and self-perceived benefit assessed with the Abbreviated Profile of Hearing Aid Benefit, and a custom questionnaire were documented. Self-perceived benefit results were compared with those for unaided listening and to ratings with participants’ own acoustic hearing aids.

**Results:**

The prescribed low-level insertion gain from 6 to 10 kHz averaged 53 dB across all ears, with a range from 26 to 86 dB. After adjustment, the gain from 6 to 10 kHz decreased to an average of 45 dB with a range from 16 to 86 dB. Measured FG averaged 39 dB from 6 to 10 kHz with a range from 11 to 62 dB. Abbreviated Profile of Hearing Aid Benefit results revealed a significant improvement in communication relative to unaided listening, averaging 28 to 32 percentage points for the background noise, reverberation, and ease of communication subscales. Relative to participants’ own hearing aids, the subscales ease of communication and aversiveness showed small but significant improvements for Earlens ranging from 6 to 7 percentage points. For the custom satisfaction questionnaire, most participants rated the Earlens system as better than their own hearing aids in most situations.

**Conclusions:**

Participants used and reported subjective benefit from the Earlens system. Most participants preferred slightly less gain at 6 to 10 kHz than prescribed for experienced users by CAM2, preferring similar gains to those prescribed for inexperienced users, but gains over the extended high frequencies were high relative to those that are currently available with acoustic hearing aids.

## Abstract 2

Supplemental Digital Content is available in the text.

## INTRODUCTION

Most conventional hearing aids provide useful gain for frequencies up to 4 to 5 kHz (Moore et al. 2001; Aazh et al. 2012; Struck & Prusick 2017). There has been debate over many years about the benefits of providing gain at even higher frequencies (Skinner & Miller 1983; Ricketts et al. 2008; Moore et al. 2010a, 2011; Moore & Sek 2013; Levy et al. 2015; Moore & Sek 2016a), or for the purposes of this paper, “extended high frequencies” defined as frequencies above 5 kHz. Extended high-frequency (HF) amplification may be of most benefit in complex listening environments with multiple speech and noise sources, especially when the sources are spatially distributed (Turner & Henry 2002; Plyler & Fleck 2006; Ahlstrom et al. 2009; Moore et al. 2010a; Hornsby et al. 2011; Levy et al. 2015). The mechanisms that may lead to improvements include the ability to take advantage of head-shadow effects, which increase markedly at HFs (Moore et al. 2010a; Levy et al. 2015), and better perception of the spatial locations of sound sources, based partly on the use of pinna cues (Best et al. 2005), which are heavily dependent on HF information.

Studies on the usefulness of amplification at HFs have differed in the definition of HF, the types of participants, the specific methods used, and the outcome measures used, leading to mixed conclusions about the potential usefulness and profile of users who may benefit from extended HF amplification (Murray & Byrne 1986; Rankovic 1991; Hogan & Turner 1998; Vickers et al. 2001; Simpson et al. 2005; Ricketts et al. 2008; Füllgrabe et al. 2010; Moore et al. 2010a, 2011). Until recently, there was no published method for prescribing gain up to 10 kHz. The most widely used hearing aid fitting methods give gain recommendations for frequencies up to 6 or 8 kHz (Byrne & Dillon 1986; Cornelisse et al. 1995; Scollie et al. 2005; Keidser et al. 2011). The Cambridge Method for Loudness Equalization 2 - High Frequency (CAM2) fitting method was developed to provide gain prescriptions for hearing aids that are capable of producing useful amplification for frequencies up to 10 kHz (Moore et al. 2010b). Despite the availability of a suitable fitting method, challenges arise in achieving sufficient audibility at extended HFs with conventional acoustic hearing aids, due to device limitations in bandwidth and output, and problems with acoustic feedback. For this reason, tests of the benefit of extended HF amplification have been largely limited to the laboratory. A relatively new device, the Earlens system (Earlens Corporation, Menlo Park, CA) (Fay et al. 2013; Gantz et al. 2017), has overcome this limitation. The Earlens is a light-driven contact hearing aid incorporating a transducer that directly drives the tympanic membrane (TM), which allows high gain and output over the frequency range from 125 to 10,000 Hz, reduces the risk of acoustic feedback (Levy et al. 2013; Khaleghi & Puria 2017) due to the low sound pressure levels produced in the ear canal by the vibration of the TM, and has been shown to be capable of producing the gains and output levels required by the CAM2 algorithm for frequencies up to 10 kHz (Fay et al. 2013; Puria et al. 2016).

The Earlens system allows exploration of the potential benefits of extended HF hearing for individuals with hearing loss in real-life settings. This article describes the results of initial evaluations of the fitting and efficacy of the Earlens system when using the CAM2 prescription, based on real-world use across eight sites by a large cohort of participants. Data were obtained during two prior clinical trials where the main goals were the assessment of safety and efficacy. However, additional data were collected and are used in new analyses reported here to explore the typical gains and output levels that were prescribed and achieved over the extended HFs and to report the adjustments from computed targets that were required based on participant reports after real-world field experience. Gains and output levels prescribed by CAM2 for the initial fit are compared with the corresponding levels after adjustment to align with functional gain (FG) expectations and individual participant preferences, and changes are discussed in terms of their clinical implications. In addition, self-perceived benefit outcomes and satisfaction ratings for the adjusted fittings are used to examine the relationship between subjective outcomes, programmed gain settings, and audiometric characteristics. This information is a first step towards further exploration of the possible benefits of providing extended HF audibility to hearing aid users.

## MATERIALS AND METHODS

Data for this study were collected during the course of two nonblinded clinical trials of the Earlens system that were conducted under a Food and Drug Administration (FDA) investigational device exemption. A complete description of the clinical trial and outcomes of the first study, which was intended to demonstrate the safety and effectiveness of the Earlens system to support FDA clearance, can be found in Gantz et al. (2017). A publication describing the second clinical trial, which was intended to gain experience across a broader cross-section of clinical practices and practitioners, is in progress (McElveen et al., Reference Note 1). Both studies used an interventional, single-group assignment, open-label design. They were approved by an institutional review board and were registered at ClinicalTrials.gov (NCT02042404 on 1/22/14 and NCT02470494 on 6/12/15). The first study (study 1) was conducted at three sites and enrolled 48 participants between March 2014 and March 2015. The second study (study 2) was an eight-site study—including the three sites from study 1—which enrolled 46 participants and was conducted between June 2015 and November 2016. At all sites, fitting was performed by experienced audiologists who had received training on the Earlens system. Performance with the Earlens system was assessed at various points during each study.

This article presents additional data collected during the trials and analyzes relationships between the additional data and prior efficacy data. Data are presented for unaided listening (collected before the devices were fitted), aided results at first fit or 1 week post-first fit, and aided results collected at the nominal end point of each study: at 90 days (study 2) or 120 days (study 1). FG data from study 1 have previously been reported as a secondary endpoint from testing at the 30-day interval (Gantz et al. 2017). FG data from study 2 for the 90-day test interval will be available shortly (McElveen et al., Reference Note 1). Subsequent to the initiation of these studies, clearance was obtained from the FDA, and the Earlens system is now available commercially.

### Participants

**Fig. 1.**
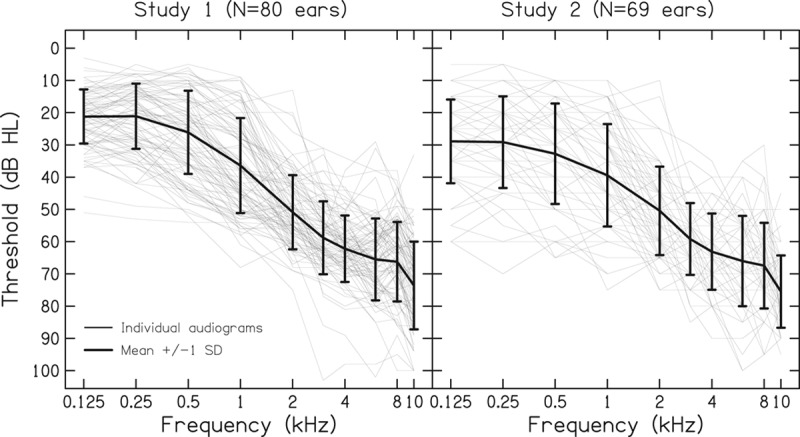
Baseline air conduction thresholds measured through earphones for the ears analyzed in the current study: 80 ears in study 1 (left panel) and 69 ears in study 2 (right panel). See the last paragraph of the Procedures section for detail regarding participant data included in these analyses. The averages are represented by the thick solid black lines with error bars representing ±1 SD.

Participants were recruited from various sites using institutional review board–approved materials, including from nearby private audiology practices, via existing patients of Earlens Corp, and by word-of-mouth. They were required to meet the medical and anatomical criteria for wearing the Earlens system. Those enrolled were between 33 and 84 years old, with an average of 68 years. All participants were fluent speakers of American English, and all but four were native speakers of American English. Participants had mild to severe sensorineural hearing loss in one or both ears (see Fig. 1), suprathreshold word recognition scores (NU-6 word lists) of 60% or better, and normal tympanometry (type A). Eighty-two percent of participants (77 of 94) had prior experience with air conduction hearing aids. All 48 participants in study 1 had bilateral hearing loss and were fitted with devices bilaterally. Five of the 48 participants (10%) discontinued use of the device before the 120-day endpoint, four due to unrelated adverse events, and one due to an inability to meet the study time requirements. In study 2, 44 of 46 participants had bilateral hearing loss and were fitted bilaterally. Of the two participants fitted unilaterally, one had normal hearing in the opposite ear and the other had bilateral hearing loss, but only one ear met the anatomical criteria for the Earlens system. Five of the 46 participants (11%) discontinued use of the device before the 90-day endpoint. Reasons included autophony (n = 2), claustrophobic sensation (n = 1), Parkinson’s condition present before enrollment (n = 1), and itchiness in the ears (n = 1).

### Materials

Audiological evaluations were conducted using the study site’s clinical equipment (audiometer, transducers, and tympanometer). Equipment models varied by site, but all had been calibrated within the past year, including frequency-specific stimuli from 9 to 10 kHz for audiometers with testing capabilities at those frequencies. The audiometer was used to present pure-tone signals for air- and bone conduction threshold measurements, recorded NU-6 word lists (Auditec, short interstimulus interval), and warble tones for sound field threshold measurements. Self-perceived benefit was assessed with the Abbreviated Profile of Hearing Aid Benefit (APHAB) questionnaire (Cox & Alexander 1995), a validated measure commonly used to assess hearing aid benefit that yields mean values for a number of subscales, as well as a global average. Satisfaction was assessed using a questionnaire specifically designed for these studies, called the Earlens Satisfaction Questionnaire. The satisfaction questionnaire was lengthy and included many questions not relevant to the current study. The current analysis was limited to questions related to satisfaction, perceived benefit, and preference (see Document, Supplemental Digital Content 1, http://links.lww.com/EANDH/A476, which details the questions and response scales for each study that are discussed in the current article). Responses were made on an ordinal 5- or 6-point Likert scale spanning a range from negative to positive, with each point labeled with a descriptor. The satisfaction questionnaire was revised between the two studies; individual questions were updated, and the response scale was changed to allow for a neutral response at the center of the scale.

Subjective comparisons of Earlens to participant’s own air conduction hearings aids are reported for the APHAB and some items on the satisfaction questionnaire. Participants’ own hearing aids were evaluated in the “as is” condition as these studies were not intended primarily as comparison studies but rather to establish the safety and efficacy of the Earlens system.

### Study Device

**Fig. 2.**
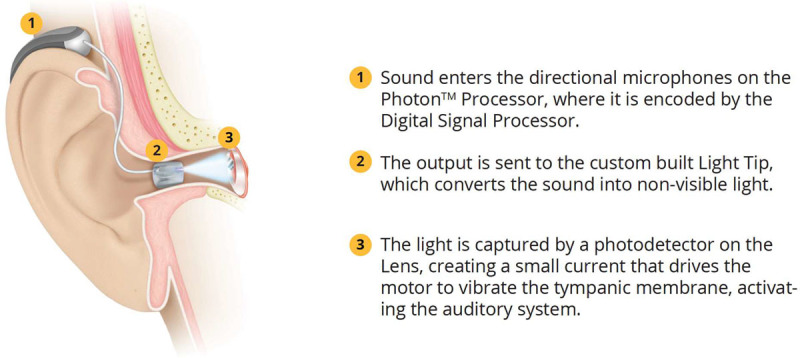
Illustration of the main components of the Earlens system.

As illustrated in Figure 2, the Earlens system consists of three components: the photon processor, a widely vented custom light tip, and the tympanic lens. The processor is similar in function to the behind-the-ear component of a receiver-in-the-canal hearing aid. The light tip is similar to a custom receiver-in-the-canal shell except that it holds a laser diode instead of a receiver. The lens is composed of a microactuator mounted on a custom ring-shaped support platform that rests on the skin at the base of the external ear canal. A photodetector receives the emitted light signal from the laser in the light tip, converting it into an electrical current that in turn activates the microactuator, which mechanically drives the TM at the umbo. In this situation, the sound radiation out of the ear canal due to vibration of the TM decreases with increasing frequency due to the presence of multiple vibrational modes (Fay et al. 2006), thus producing less feedback than for an equivalent level of an acoustic sound source in the ear canal (Puria 2003).

The processor includes a 20-channel compression system, with an attack time of 50 ms and a release time of 1000 ms in each channel. A constant compression ratio is applied for levels between the compression threshold (CT) and the level at which compression limiting occurs, which is referred to as the maximum equivalent sound pressure output (MPO), similar to the MPO for acoustic hearing aids. Expansion is applied for levels more than about 6 dB below the CT. Fast-acting compression limiting is applied for output levels above the output compression limiting threshold in each channel. A feedback cancellation system can be activated to achieve additional stable gain if required. The processor is programmed using Hi-Pro 2 interface hardware and proprietary software that is similar in function to that for acoustic hearing aids, but with two additional features: a light calibration procedure and FG mode, described later.

### Procedures

Participants were enrolled after agreeing to be included in the study via standard informed consent procedures. The following measures were obtained using standard clinical procedures for each ear to determine whether the candidacy criteria were met: air conduction and bone conduction audiometric thresholds (using 2-dB steps for study 1 and 5-dB steps for study 2); word recognition scores for recorded NU-6 word lists at the presentation level giving the highest score, PBmax (50-word list for study 1 and 25-word list for study 2); and tympanometry. Baseline unaided sound field thresholds were measured for each ear with the nontest ear plugged or masked as needed,* using a loudspeaker calibrated at 0° azimuth and elevation. The APHAB (Cox & Alexander 1995) questionnaire was administered for baseline unaided listening and for listening via the participants’ own air conduction hearing aid(s) (if applicable).

After placement of the tympanic lens, a calibration procedure was performed for each ear to measure the effective output in response to a calibrated light signal as a function of frequency. For this purpose, sinusoidal signals were synthesized and delivered as pulse-density modulated light. The intensity of the light required for the signal to be detected was measured for each frequency. We denote the light intensity at threshold for a given frequency as I_f_. The previously measured audiometric threshold at each frequency in dB HL was converted to the equivalent in dB SPL, denoted T_f_. In this way, it was known that a light intensity of I_f_ would give a sound level equivalent to T_f_. This procedure is conceptually equivalent to calibration of an air conduction hearing aid using a probe microphone in the ear canal although the uncertainty of the calibration at HFs is much less for the light-calibration procedure than for probe-microphone measurements due to the absence of ear-canal standing waves that affect probe-microphone measurements. The light-calibration procedure combined with the maximum possible value of intensity of the light was used to determine the maximum possible equivalent sound pressure output (MPPO) of the system on each ear at each frequency. This is different from the MPO, mentioned earlier, as the MPO was adjustable for each ear in the fitting software; the MPO was always equal to or below the MPPO. After the system was calibrated in this way, participants were fitted with the CAM2 prescription for frequencies up to 10 kHz, using the Earlens fitting software. All participants were initially fitted with the “full” gains recommended by CAM2 for experienced users of hearing aids. Gains were then either manually adjusted or automatic acclimatization (when available) was enabled based on informal participant feedback. Recent versions of the CAM2 software incorporate reduced gains for inexperienced users of hearing aids (Moore & Sek 2013, 2016a), but the version used for the studies reported here did not incorporate this option.

The fittings were adjusted to provide comfort and to reduce acoustic feedback when necessary. In study 1, only minimal adjustments were made for feedback before measurement of FG at the first fit visit (these program settings are termed “First Fit Initial,” see later for details), but more substantial adjustments were sometimes made after measurement of FG at the first fit visit, especially when the FG appeared to be markedly lower or higher than expected from the programmed gains (these adjusted program settings are termed “First Fit Adjusted”). In study 2, no First Fit Initial measurements were made as the study was designed to more closely mimic normal commercial flow, and therefore adjustments were permitted before the measurement of FG (these measurements are termed “First Fit Adjusted”). In study 2, acclimatization became available in the fitting software mid-study, and it was activated for one third of the participants. When activated, the programmed gains were initially set below the target values, either for all frequencies or only for the extended HFs (6 to 10 kHz). The gains were then gradually increased towards the target values over a period of between 1 and 6 weeks, depending on the autoacclimatization settings chosen by the audiologist.

Aided sound field thresholds were measured for each ear as described for the unaided condition, by placing the device in a “functional gain mode” which programs the device to be linear (no compression) and sets the insertion gain (IG) in each channel to the IG at the Compression Threshold (IGCT) for the current fitting. The IGCT represents the maximum IG that the device would apply at each frequency, and it depends on both the hearing level of the individual and the spectrum of “soft” (50 dB SPL) speech. FG at each frequency was calculated as the difference between the baseline unaided sound field threshold and the aided sound field threshold. Measures of FG in the sound field were compared with the prescribed and programmed IGs at multiple time points to “verify” the fitting as real ear measures are not possible with this nonacoustic device.

The APHAB questionnaire for the aided Earlens condition was administered at 90 or 120 days post-fitting. The Earlens-designed Satisfaction Questionnaire was also administered at 90 or 120 days post-fitting. The programming software fitting files were saved at each time point so that prescribed and adjusted (current fit) output and gain data could be extracted and analyzed.

Not all subjects completed all measurements at all test intervals. Five participants who did not complete each study were removed from the current analysis. In addition, among those who completed the study, there were a few instances of incomplete data sets, usually due to missing aided sound field data at first fit or at the endpoint of the study, or occasionally a missing fitting file. These instances occurred across multiple test sites. Thus, data from six additional ears were removed from the output and gain analyses of study 1, and 11 additional ears were removed from the same analyses for study 2. Therefore, the current analysis includes 42 participants (80 ears) for study 1 and 36 participants (69 ears) for study 2 with complete study data. In the case of APHAB and satisfaction data, which are participant-based instead of ear-based, the data of these participants were also removed, including those fit bilaterally but with complete data in only one ear. In addition, subjects who wore their own hearing aids before the study were also tested with those aids, leading to different numbers of subjects in comparisons of Earlens to unaided and Earlens to own hearing aid. All comparisons that were analyzed statistically used paired-comparison methods based only on participants with data for both variables involved in the comparison, and only those participants’ data are plotted.

## RESULTS

### Output Levels

The data extracted from the fitting software include effective output level in dB SPL for “soft” (50 dB SPL), “moderate” (65 dB SPL), and “loud” (80 dB SPL) input levels of speech. The effective output level at each center frequency was calculated by integrating the speech spectrum power across one equivalent rectangular bandwidth (ERB) of the auditory filter centered at that frequency. The value of the ERB for each participant and center frequency was estimated taking into account the hearing loss of the participant at that center frequency, as described by Moore and Glasberg (2004). Audibility can be assessed by comparing the effective output level to the audiometric threshold expressed in dB SPL. The fitting data also include the MPPO and MPO. The MPO is the output compression limiting threshold and is clinician-adjustable at each channel center frequency based on individual needs. The MPO could be set equal to or below the MPPO in study 2. The MPO adjustment was not available in study 1 and therefore the MPO was equal to MPPO in that study.

**Fig. 3.**
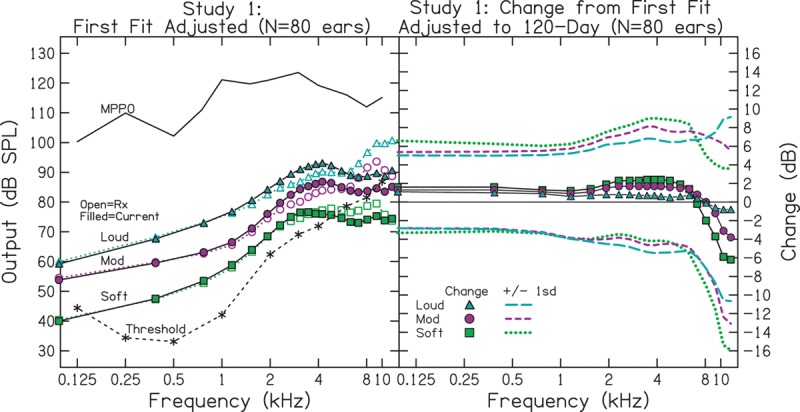
The left panel shows mean output levels and thresholds for study 1 at the First Fit Adjusted time point. The maximum possible equivalent sound pressure output is indicated by the solid line near the top of the panel. Soft, moderate, and loud outputs are indicated by squares, circles, and triangles, respectively; open symbols/dotted lines indicate the prescribed (Rx) fit, and filled symbols/solid lines indicate the current (adjusted) fit. Pure-tone thresholds in dB SPL are indicated by the asterisks/dashed lines. The right panel shows the change in output between the first fit adjusted and 120-day time points (120-day minus First Fit Adjusted), with corresponding ±1 SD curves.

Figure 3 shows the mean output-level data for study 1. The left panel shows the First Fit Adjusted output curves together with the mean audiometric thresholds, converted from dB HL to dB SPL using the minimum audible pressure values for monaural listening (Moore & Glasberg 2007). Average MPPO values ranged between 100 and 124 dB SPL. Prescribed (Rx) and current fit curves for soft, moderate, and loud speech input levels are shown. The differences between the prescribed and current fit curves indicate the average adjustments made by the audiologists who fitted the devices. On average, these included a slight decrease in output for the extended HFs (6 to 10 kHz) and a slight increase in output over the range 2 to 4 kHz. The adjustment for the extended HFs was occasionally due to minor problems with acoustic feedback but more often due to complaints that the sound was harsh or that some sounds, such as /s/, were overemphasized, which was at least in part due to suboptimal output limiting that was used at the time of the study. The adjustments in the 2 to 4 kHz region were a result of FG testing, which often revealed a need for slightly increased output over that frequency range. Generally, the output was increased slightly over the range 2 to 4 kHz when the FGs in that range were more than 5 dB less than the current fit settings for IGCT or when aided sound field thresholds were poorer than 30 dB HL. FG could have differed from IGCT values due to FG measurement error (unaided threshold, aided threshold, or both) or error in threshold measurement during light calibration, and also due to the fact that the prescribed IGCT values were based on diffuse-field incidence whereas the sound field measurements were made only for sounds with a frontal incidence; this last point is discussed in more detail later. The effective output levels are expressed as root-mean-square (RMS) values. The effective dynamic range of speech extends from about 15 dB below the RMS level to 15 dB above the RMS level (ANSI 1997; Moore et al. 2008). Hence, when the RMS level of the speech at a given center frequency equals the absolute threshold at that frequency, roughly half of the effective dynamic range of speech is audible. Even after adjustments to reduce acoustic feedback and improve sound quality, the left panel of Figure 3 shows that at least partial audibility was achieved for the medium and high levels of speech for frequencies up to 8 to 10 kHz. For soft speech, at least partial audibility was achieved for frequencies up to 4 kHz.

The right panel of Figure 3 shows the average change in current fit settings from First Fit Adjusted to 120-day time points with a positive value indicating that the output was higher at 120 days than at first fit, along with the SD of the changes. While slight changes in mean output settings occurred over time between First Fit Adjusted and the 120-day visit, the settings at the end of the study were, on average, largely similar to those for First Fit Adjusted. One difference was that at the 120-day time point, on average, the output levels for the extended HFs were slightly lower for the moderate and soft speech levels. This was mainly the result of a change in fitting software parameters for the extended HFs which increased the programmed CT, keeping the gain at levels above the CT unchanged, following the recommendations of Moore and Sek (2016a). The SDs show that many of the individual changes were within ±4 to 10 dB of the average change, with the larger variability occurring in the extended HFs.

**Fig. 4.**
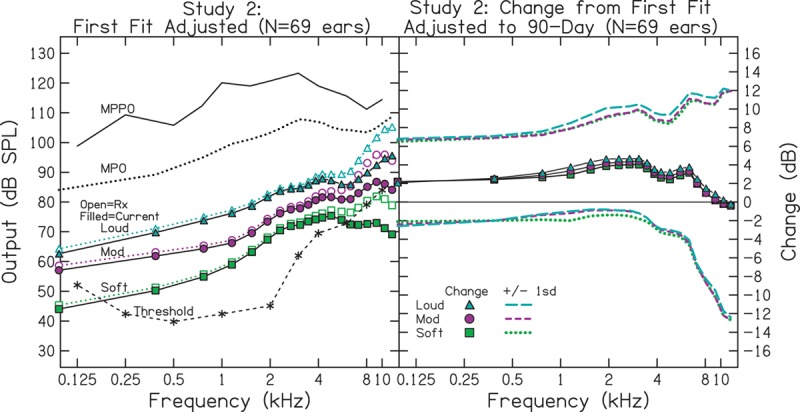
As Figure 3, but for the First Fit Adjusted (left panel) and change from First Fit Adjusted to 90-day (right panel) settings for study 2. Maximum equivalent sound pressure output (MPO) values are shown here as the dotted line with no symbols just below the maximum possible equivalent sound pressure output (MPPO) curve since the fitting software was changed to allow the MPO to be set below the MPPO for study 2.

Figure 4 is the same as Figure 3 but shows data for study 2. There are two main differences compared with study 1. First, the MPO could be adjusted for study 2, which resulted in the mean MPO being significantly lower than the mean MPPO. As mentioned earlier, this happened because after study 1 was completed, the Earlens fitting software was updated to prescribe MPO values, and the clinician was permitted to adjust MPO values as needed, both of which were designed to increase comfort for intense sounds and to reduce power consumption, hence extending battery life. Mean MPPO values ranged from 99 to 123 dB SPL while mean MPO values ranged from 84 to 109 dB SPL. The second difference is not obvious from the figures, but automatic acclimatization became available in the fitting software approximately half way through study 2. Approximately 32% of the participants in study 2 had automatic acclimatization turned on at first fit, which resulted in decreased gains at the initial fitting. Thus, the mean adjusted outputs in Figure 4 reflect a combination of the audiologist manually decreasing the outputs for the extended HF as needed and the enabling of automatic acclimatization. There were two acclimatization options: one for experienced hearing aid users, which led to reduced initial output only over the range 6 to 10 kHz; and the other for new hearing aid users, which led to reduced output across the entire frequency range but with greater reduction for the extended HFs than for lower frequencies. Most (9 of 14) participants with acclimatization activated received acclimatization only for the extended HFs.

For study 2, the 90-day time point was the nominal end of the study. Despite automatic acclimatization, which was complete before 90 days, the 90-day output values were only slightly higher than the First Fit Adjusted values for the extended HFs (see right panel of Fig. 4). The small changes between First Fit Adjusted and 90-day probably reflect the fact that only 32% of participants had acclimatization turned on. There was also a small increase in output level for all lower frequencies by 90 days, either due to the acclimatization or to manual increases made by the audiologist. The variability of the changes was similar to that seen for study 1.

**Fig. 5.**
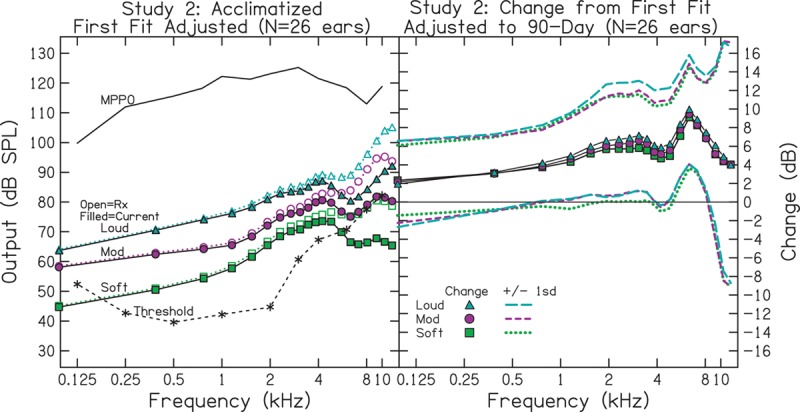
As Figure 4, but for a subset of participants from study 2 with automatic acclimatization turned on. The change in current fit curves in the extended high frequencies between First Fit Adjusted and 90-day, largely reflects the change in output as a result of turning acclimatization on. By 90 days, the acclimatization was complete.

To illustrate the effect of acclimatization more clearly, data for the 14 participants who had acclimatization activated were analyzed separately. Mean data are plotted in Figure 5 (First Fit Adjusted in the left panel and the change in current fit settings by 90 days in the right panel). For 9 of the 14 participants whose data are shown in this figure, acclimatization only affected the output levels for the extended HFs. For the remaining five participants, acclimatization affected the gain for all frequencies. The output levels at HFs increased after acclimatization, by 4 to 10 dB. However, even after acclimatization was complete, output levels for the extended HFs remained 5 to 10 dB below the values prescribed by CAM2. This reflects the combined effects of occasional output reductions to reduce acoustic feedback and output reductions to improve sound quality or comfort based on reports of the participants. However, for frequencies below 4 kHz, the mean current fit outputs at 90 days were slightly above the values prescribed by CAM2. This reflects deliberate adjustments made by the audiologists because acclimatization would not lead to output levels above the prescribed values. These adjustments were partly based on FG measurements, which sometimes indicated a need for a slight increase in output, but may partly reflect occasional participant requests for more audibility.

### Gain Values

As described earlier, the IGs programmed by the software were verified by FG measurements in the sound field (baseline unaided thresholds minus aided thresholds with the device activated in FG mode).

#### Measured FGs and Comparison to IGCT

The measures of FG at each test frequency should be closely related to the values of IGCT programmed in the fitting software. However, the two will not be numerically identical. The measures of FG were obtained in sound-treated rooms using a loudspeaker with 0° azimuth and elevation, thus approximating free-field listening conditions. However, the IGs prescribed by CAM2, including the maximum insertions gains, IGCT, are based on diffuse-field listening (Moore et al. 2010b). The two can be made comparable by correcting the FGs by the difference between the free-field-to-hearing aid microphone response and the diffuse-field-to-hearing aid microphone response (Bentler & Pavlovic 1989); these differences are given in Table 1. The CAM2-prescribed IG at the CT (Rx IGCT) is the maximum prescribed IG at a given center frequency. As the input level is increased above the CT, the IG decreases due to compression. When adjustments are made to the output or gain in the fitting software, the IGCT changes accordingly, resulting in a current fit IGCT. The current fit IGCT is similar to the IG for a soft speech input (within 1 dB), so the figures showing gains display only the IG for soft speech (filled squares) and not the current fit IGCT.

**TABLE 1.**
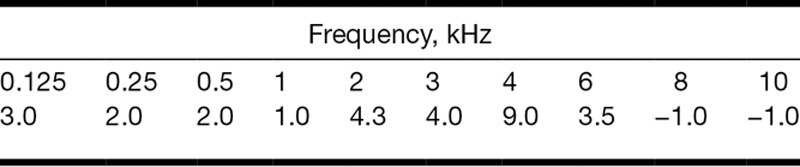
Correction factors in dB applied to functional gain data to allow for the difference between the free-field listening conditions used to gather these data and the diffuse-field conditions assumed in the Cambridge Method for Loudness Equalization 2 - High Frequency (CAM2) software

**Fig. 6.**
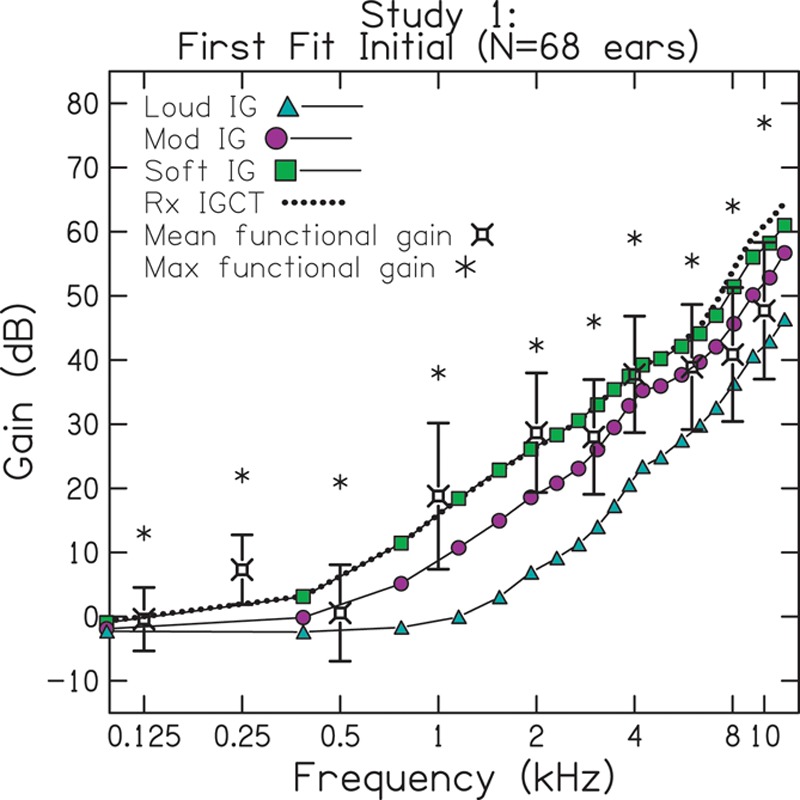
Insertion gain (IG) values for study 1 as a function of frequency for First Fit Initial settings. The dotted line shows the mean Cambridge Method for Loudness Equalization 2 - High Frequency (CAM2)-prescribed IG at compression threshold (IGCT). The solid squares, circles, and triangles show the mean current fit IGs for soft, moderate, and loud inputs, respectively. The open squares with four points and error bars show the mean functional gains ±1 SD, and the asterisks show the maximum functional gain for each frequency. Note that the sample size differs from that for the study 1 output graph; see text for explanation.

Figure 6 shows gain data for study 1, for the First Fit Initial time point. The figure shows the mean prescribed (Rx) IGCT (dotted line) and the mean current fit soft, moderate, and loud IG curves. Means and maximum values of the FG at each frequency are also shown. First Fit Initial (as opposed to first fit adjusted) data were chosen for the gain view because the corresponding FG data were measured using First Fit Initial settings, before adjustments due to any reason other than for feedback. Note that the sample size (N = 68) differs from the output data in Figure 3 (N = 80). The First Fit Initial files were occasionally over-written by the First Fit Adjusted file created on the same day, and as a result, the First Fit Initial programmed data were not available for 12 ears. Thus, to be conservative, those ears were excluded from this comparison. However, when compared with the full set of 80 ears, the FG data for the 68-ear subset were within 1 dB of those for the full set at each frequency. Comparing the Rx IGCT to the soft IG curve reveals that gain adjustments needed for feedback were on average quite small, between 1 and 3.5 dB at 6 to 10 kHz. The First Fit Initial FG followed the current fit soft gain curve fairly well up to 4 kHz. From 6 to 10 kHz, the mean FG fell below the soft IG by 6 to 11 dB. The small differences between the mean FG and the soft gain settings may reflect small errors in the calibration of the microphones in the photon processor, errors of measurement, and (for low frequencies [LFs]) the fact that free-field listening conditions were only approximately achieved. Another possibility is that because the programmed gain for soft sounds was often relatively high at HFs, the output level sometimes reached the MPO, restricting the output and thus the FG to a value below what was programmed. However, the maximum FG was remarkably high, being 77 dB at 10 kHz. The corresponding changes in gain settings from first fit to 120-day time point are not shown because the changes in output shown in the right panel of Figure 3 closely mirror the changes in gain (within 0.5 dB). Table 2 shows the changes in FG between the two time points. For study 1, the average FG was lower at 120 days than at First Fit Initial. For the extended HFs, this was at least partly the result of audiologist adjustments. However, for lower frequencies, FG was also slightly lower at 120 days than at first fit despite a slight increase in programmed settings (right panel of Fig. 3). The reason for the increased mismatch between programmed settings and FG at 120 days is unclear, but potential explanations include decreased microphone outputs in the photon processor over time because of the accumulation of dirt, cerumen, and other materials on the diaphragms of the microphones or around the microphone ports or a change in the light calibration due to poor light tip placement for FG testing or other mismatch in the relationship between the light tip output and the effective acoustic output of the lens. Fitting data showed that at least 70% of ears received an updated light calibration at the 120-day visit, making the last explanation less likely.

**TABLE 2.**
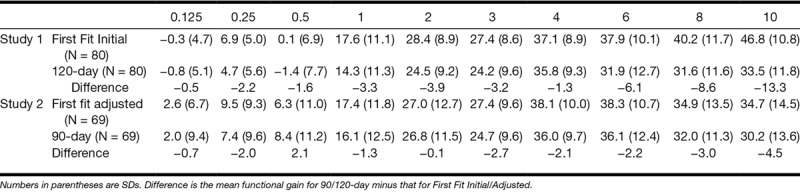
Mean functional gain in dB at first fit and study endpoint for study 1 and study 2

**Fig. 7.**
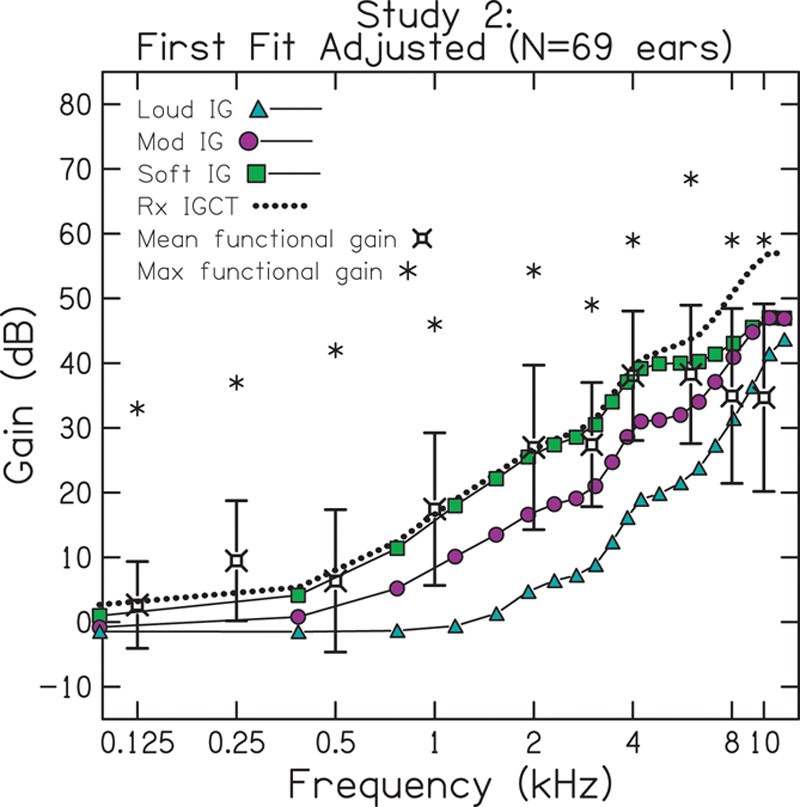
As Figure 6, insertion Gagin (IG) and functional gain values for study 2 as a function of frequency for First Fit Adjusted settings. The dotted line shows the mean CAM2-prescribed IG at compression threshold (IGCT). The solid squares, circles, and triangles show the mean current fit IGs for soft, moderate, and loud inputs, respectively. The open squares with four points and error bars show the mean functional gains ±1 SD, and the asterisks show the maximum functional gain for each frequency.

The First Fit Adjusted gain values for study 2 are shown in Figure 7. The maximum FG was 69 dB at 6 kHz. The FG curve at first fit followed the IG curve for soft speech, as expected, except at 8 to 10 kHz where it fell below the programmed soft speech IG by about 10 dB. This was also seen at First Fit Initial in study 1 (Fig. 6) and may similarly reflect errors in calibration, errors in threshold measurement, or output limiting due to the high programmed gains for soft sounds. Table 2 shows the FG at the 90-day endpoint compared with First Fit Adjusted for study 2. Relative to study 1, there was a similar but smaller decrease in FG at 90 days compared with first fit, particularly at 8 to 10 kHz. The decrease over the extended HFs was smaller in study 2 than study 1 because in study 2, the first fit FG was measured after audiologist adjustments whereas in study 1 it was measured before audiologist adjustments to gain (except for feedback). Although the programmed settings were adjusted up by 3 to 4 dB at 1 to 6 kHz (right panel of Fig. 4), FG demonstrated a slight drop or no change in the same frequency range. Again, the reasons for the discrepancies are not clear and may reflect any of the factors described earlier, including changes in microphone sensitivity.

#### Gains After Audiologist Adjustments

**Fig. 8.**
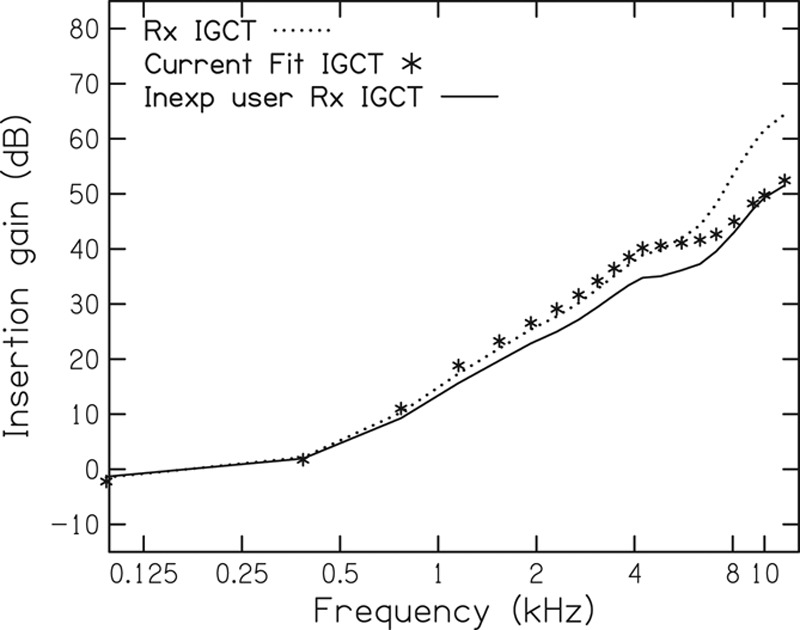
Comparison between CAM2-prescribed insertion gain at compression threshold (IGCT; dotted line), CAM2-prescribed inexperienced user IGCT (solid line), and the current fit IGCT (*) as a result of audiologist adjustment to the fitting at first fit.

The current version of the CAM2 software includes a modified prescription for inexperienced hearing aid users (Moore & Sek 2013, 2016a). Although most of the participants in the current study were experienced hearing aid users, they had not previously experienced amplification over the extended HF range (6 to 10 kHz), so, at least for that frequency range, the gains recommended for inexperienced users might be more appropriate. To assess this, we compared the CAM2 inexperienced-user IGCT values to the current fit IGCT values obtained after the adjustment at first fit, to see if the adjustments that the audiologists made led to IGs similar to those prescribed by CAM2 for inexperienced users. The outcomes for study 1 are shown in Figure 8 (the same results were obtained for study 2, and thus the data are not shown here). Over the range 6 to 10 kHz, the current fit IGCT values (asterisks) were indeed consistent with the IGCT values recommended for inexperienced users (solid line). For lower frequencies, the current fit IGCT values matched the CAM2 standard prescription (dotted line) more closely. This was expected because most participants were previous hearing aid users and therefore were experienced with amplification at 4 kHz and below; they were only inexperienced with amplification over the range 6 to 10 kHz.

### Self-Perceived Benefit

#### APHAB Questionnaire

**Fig. 9.**
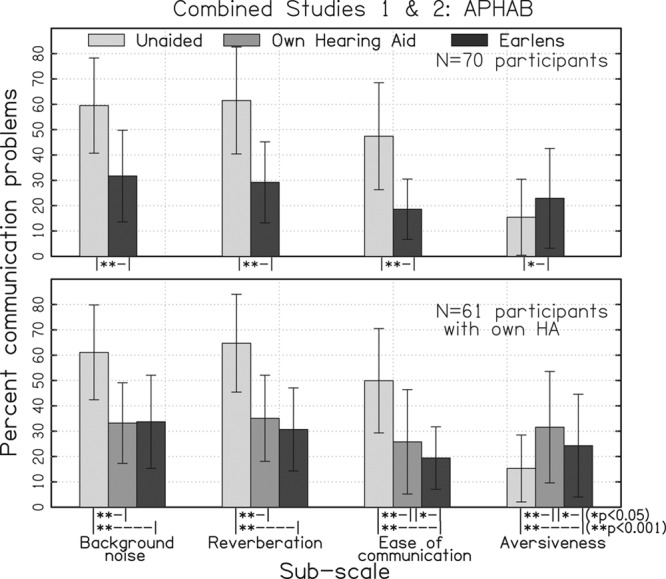
Abbreviated Profile of Hearing Aid Benefit (APHAB) scores showing mean percent of self-perceived communication problems for each of the subscales. Light gray bars are for the unaided condition, medium gray bars are for the participants’ own hearing aids, and black bars are for aided with Earlens at the 90- or 120-day time point. The upper panel is for the comparison of Earlens to unaided listening while the lower panel shows data for the subset of participants with their own hearing aids. Error bars show ±1 SD. Asterisks indicate significant differences at *p* < 0.05 or *p* < 0.001.

The APHAB questionnaire was administered at baseline before fitting for unaided listening and to assess experiences with the participants’ own hearing aid(s), which applied to most participants. Experience in listening with the Earlens system was evaluated at the end point of each study. APHAB data were pooled between the two studies for this analysis to give increased statistical power. The upper panel of Figure 9 shows the mean results for each subscale for the Earlens-unaided comparison. For these comparisons, the power was sufficient to detect a difference of between 6 and 7 percentage points 80% of the time with a *p* value of 0.05. A two-factor repeated-measures analysis of variance was performed with the factors: listening condition (unaided, Earlens) and subscale (background noise, reverberation, ease of communication, aversiveness). There was a significant main effect for both listening condition [*F*(1, 69) = 187.2, *p* < 0.001] and subscale [*F*(3, 207) = 90.2, *p* < 0.001], and the listening condition × subscale interaction [*F*(3, 207) = 83.7, *p* < 0.001]. Communication problems for the background noise, reverberation, and ease of communication subscales decreased by 28 to 32 percentage points when fitted with Earlens relative to the unaided condition. Sidak multiple comparisons test revealed that these differences were significant [*t*(207) = 13.6 to 15.8, *p* < 0.001)] for each comparison. For aversiveness, there was a significant increase of 7.5 percentage points for Earlens relative to unaided [*t*(207) = 3.68, *p* < 0.01]. The finding for aversiveness is not unusual as many studies of hearing aids have shown an increase in aversiveness relative to unaided listening (Moore et al. 2005; Moore & Popelka 2013). The lower panel of Figure 9 shows comparisons between unaided, own hearing aid, and Earlens conditions. For these comparisons, the power was sufficient to detect a difference of 7 percentage points 80% of the time with a *p* value of 0.05. A two-factor repeated-measures analysis of variance was performed with the factors: listening condition (unaided, own HA, Earlens) and subscale (background noise, reverberation, ease of communication, aversiveness). There were significant main effects of listening condition [*F*(2, 120) = 97.2, *p* < 0.001], subscale [*F*(3, 180) = 50.5, *p* < 0.001], and the interaction [*F*(6, 360) = 60.3, *p* < 0.001]. Tukey’s multiple comparisons tests revealed that relative to the unaided condition, there was a significant decrease in communication problems on the background noise, reverberation, and ease of communications subscales for both Earlens and own HA (*p* < 0.001 in all cases). Also, aversiveness increased for both own HA and Earlens relative to unaided (*p* < 0.001 for each), by 16 percentage points for own HA and by 9 percentage points for Earlens. The difference between own hearing aids and Earlens was not significant for background noise [*q*(360) = −0.29, *p* = 0.977], or reverberation [*q*(360) = 2.83, *p* = 0.113], but was significant for ease of communication [*q*(360) = 4.06, *p* < 0.05] with an average decrease of problems of 6.4 percentage points for Earlens. There was also a difference for aversiveness [*q*(360) = 4.66, *p* < 0.01], with Earlens showing an average decrease in problems of 7.3 percentage points relative to own HA.

#### Patient Satisfaction Questionnaire

The specific questions for which the responses were analyzed are contained in the Supplemental Digital Content 1, http://links.lww.com/EANDH/A476: Relevant Excerpts of the Earlens Patient Satisfaction Questionnaires. These include questions relating to satisfaction (overall, listening benefit, and sound quality) and perceived benefit relative to unaided listening and listening with own hearing aids across a variety of situations. Statistical analyses were not performed on these data due to lack of power, particularly for study 2, and because there was no straightforward way of combining data across studies due to the different questions and response scales. In addition, the questionnaire was not validated.

**Fig. 10.**
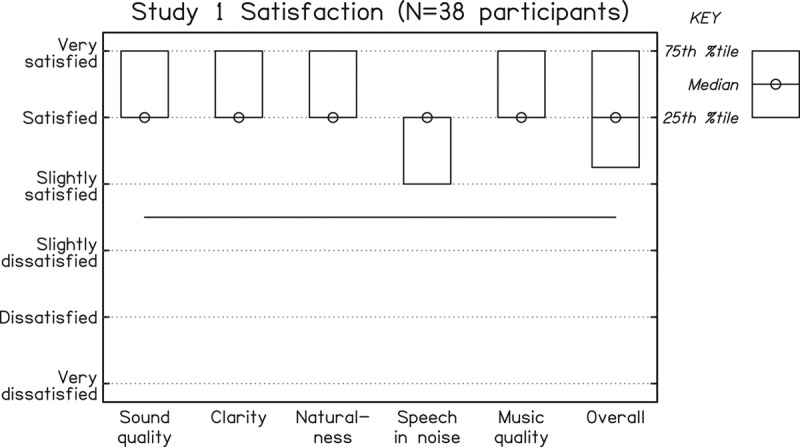
Study 1 satisfaction ratings for the Earlens system on a 6-point Likert scale (shown on the *y* axis) at the 120-day time point for the categories specified along the *x* axis. The median rating is indicated by the open circles, and the 25th and 75th percentiles are indicated by the lower and upper edges of the boxes. The solid horizontal line in the center indicates the neutral mid-point of the rating scale. Note: the sound quality and speech in noise data were previously presented in a different format in Gantz et al. (2017).

**Fig. 11.**
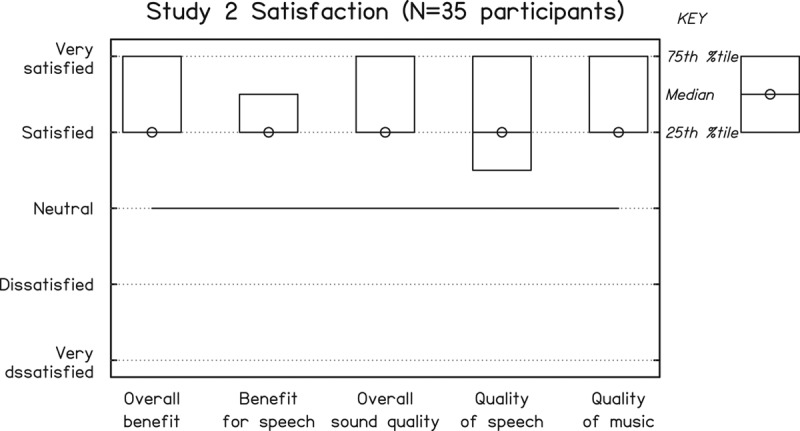
As Figure 10, but for study 2 at the 90-day time point. Satisfaction ratings were obtained on a 5-point Likert scale, indicated on the *y* axis, for the categories assessed on the *x* axis. The neutral rating is marked by the solid horizontal line. Note: data presented in a different format in McElveen et al. (Reference Note 1).

Median satisfaction ratings and upper and lower quartiles for study 1 are shown in Figure 10. The midpoint rating was in between “Slightly satisfied” and “Slightly dissatisfied” for these questions. The median satisfaction rating was “Satisfied,” with the upper quartile (75th percentile) reaching the maximum rating of very satisfied, for all categories, except for the “Speech in noise” category. For this category, the ratings tended to be lower while the lower quartile (25th percentile) remained above the mid-point at “Slightly satisfied.” Satisfaction ratings for study 2 are shown in Figure 11. For this study, the midpoint rating was “Neutral.” The median rating was “Satisfied” for all categories, with the upper quartile at “Very satisfied” for most questions. The lower quartile remained above the neutral midpoint for all categories.

**Fig. 12.**
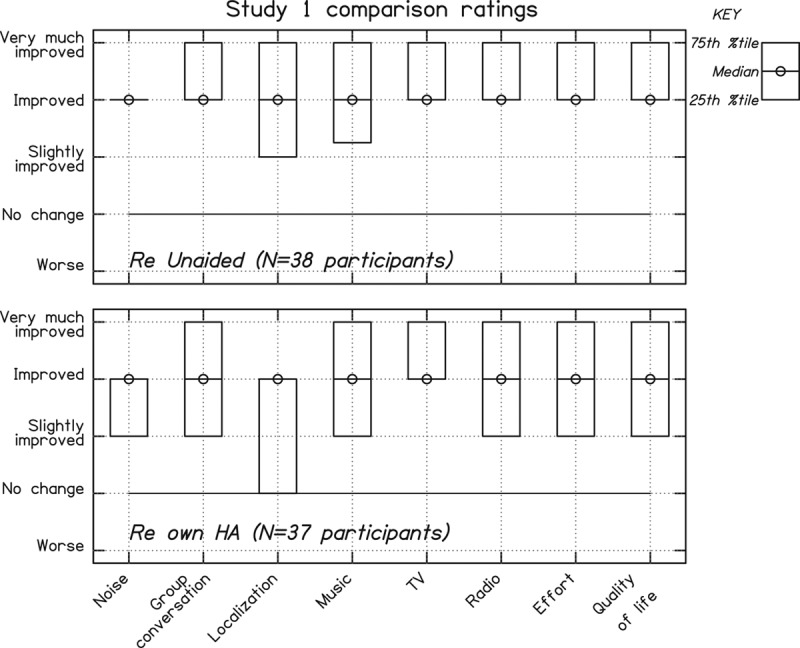
Study 1 comparison ratings for Earlens relative to unaided listening (top panel) and relative to participants’ own hearing aids (lower panel). Ratings were obtained on a 5-point Likert scale, shown on the *y* axis, for the categories shown on the *x* axis. Median ratings are indicated by the open circles, and the 75th and 25th percentiles are indicated by the upper and lower edges of the boxes. The neutral mid-point of the rating scale is indicated by “No change” and the solid horizontal line. Note: the Quality of life comparison to unaided listening only (top panel) was previously presented in a different format in Gantz et al. (2017).

**Fig. 13.**
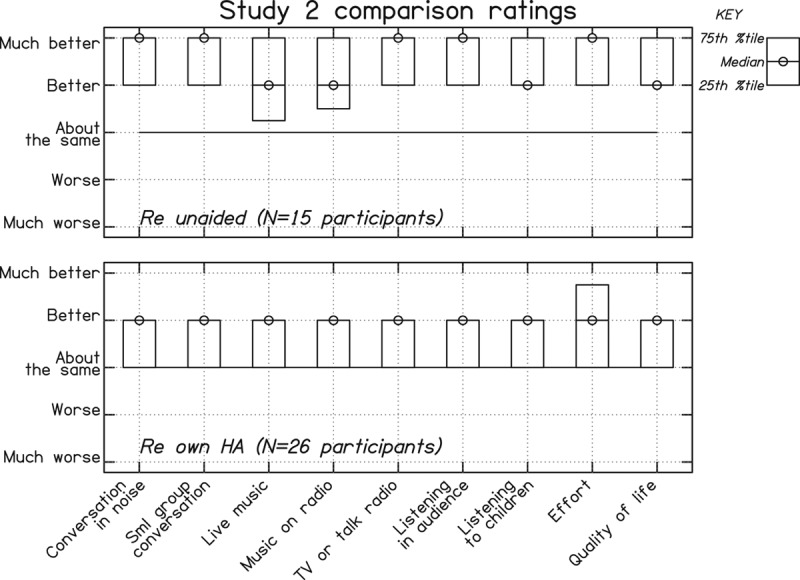
As Figure 12, but for study 2 at the 90-day time point. Satisfaction ratings were obtained on a 5-point Likert scale, indicated on the *y* axis, for the categories assessed on the *x* axis. The neutral rating is “About the same” and is marked by the solid horizontal line.

Participants were also asked how the Earlens compared with listening unaided or listening with the hearing aid(s) that they used before the study. The median and upper and lower quartiles of the responses are shown in Figure 12 for study 1 and Figure 13 for study 2. The specified listening situations are indicated on the *x* axis. The upper panel is for comparison to unaided listening while the lower panel is for comparison to the participants’ own hearing aids. For both studies, the median ratings for all listening situations were clearly above the neutral rating of “No change” or “About the same,” relative to both unaided listening and listening with their own hearing aids. Ratings compared with unaided hearing were generally better than those compared with own hearing aids, as expected. However, the results indicate that the Earlens system was generally rated as better than the participants’ own hearing aid(s). For study 1, the lower quartile was above “No change” for most listening situations while for study 2, the lower quartile of ratings reached the “About the same” rating for most categories.

### Correlations Between Objective and Subjective Outcomes

In fitting any hearing aid for a person with sensorineural hearing loss, there is often a compromise between fully matching prescriptive targets to optimize the audibility of speech sounds and meeting the user’s preferences for loudness and sound quality. In addition, most people with HF hearing loss lack experience with amplification over the extended HF region. While the Earlens system is able to provide substantial HF and extended HF gain, it was not initially clear whether users would like it and perceive benefit from it and how perceived benefit might vary with the type and amount of hearing loss. The answers to these questions can help determine best-practice gain settings for optimizing satisfaction. In addition, although the Earlens fitting range is quite wide, it is possible that individuals with certain audiometric characteristics have greater subjective benefit and preference for Earlens. This led us to conduct a correlational analysis to assess whether self-perceived benefit and satisfaction were related to measures, such as audiometric parameters of the underlying hearing loss (pure-tone average and slope), measured FG, or adjustments made to the fitting over or under prescriptive targets. There were no predetermined theories about which hearing profile might benefit most or least or whether fitting at or under targets would have a positive or negative effect on subjective ratings. The correlation was determined between each of several objective summary measures and each of several subjective summary measures. The objective measures were as follows: pure-tone average (PTA) for LF (0.125 to 1 kHz), HF 2 to 10 kHz), and broadband (broad = 0.125 to 10 Hz); slope for LF, HF, and broad; FG for LF, HF, and broad; and mismatch (the difference in output between the CAM2 prescription and the current fitting for speech with an input level of 65 dB SPL) LF, HF, broad, speech frequencies (Sp Freq = 0.5 to 4 kHz), and extended HF (6 to 10 kHz). The subjective measures were as follows: APHAB global benefit for Earlens (relative to unaided listening or listening with the participants’ own hearing aid(s), HA) averaged across the background noise, reverberation, and ease of communication subscales; and average satisfaction, perceived benefit re-unaided listening, perceived benefit re-own hearing aid(s), and preference re-own hearing aids. The last four measures were based on averages across responses from applicable questions on the Earlens Patient Satisfaction Questionnaire.

The results are shown in Table 3 for APHAB, for which the data were pooled across the two studies for increased statistical power. The power was sufficient to obtain significance at *p* < 0.05 with a correlation coefficient down to ≈0.25. Significance levels were not corrected for multiple testing as we considered this only a preliminary exploration. Even without correction, most of the correlations were not statistically significant. Only slope LF was weakly correlated with APHAB benefit re-unaided, such that as the slope increased, benefit re-unaided increased. Correlations between the same objective measures and average satisfaction ratings, perceived benefit, and preference re-own hearing aid were calculated separately for studies 1 and 2 as these data could not be easily pooled. The power was only sufficient to obtain significance for correlations above 0.32 to 0.35 due to smaller sample sizes (N = 26 to 38, depending on the study and comparison). None of the objective measures were significantly correlated with the subjective outcomes. The fact that all correlations were small indicates that the objective measures did not account for much of the variability in subjective responses. Different results might have been obtained with a larger sample size.

**TABLE 3.**
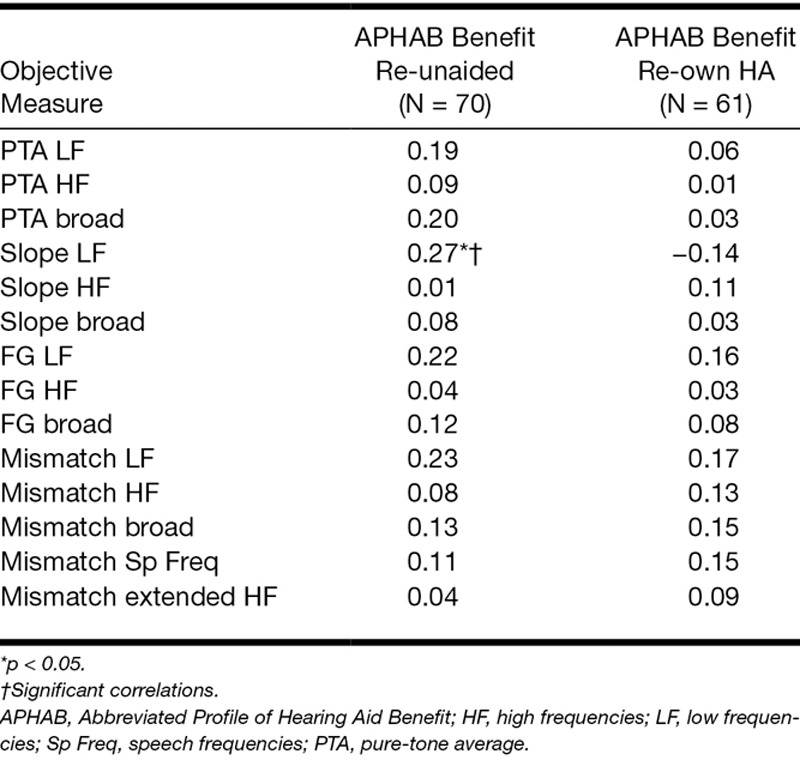
Correlation coefficients between objective and APHAB measures for studies 1 and 2 combined (N = 70/61 re-unaided/own HA)

## DISCUSSION

The Earlens system was able to provide the gains and output levels prescribed by the CAM2 fitting method for a wide range of hearing losses and over the whole frequency range up to 10 kHz. Peak output levels averaged 123 to 124 dB SPL, similar to those for the temporal bone data of Puria et al. (2016) (Figs. 3 and 4), and IGs exceeding 60 dB were possible at HFs (Figs. 6 and 7). Participants reported appreciating the audibility provided by the large prescribed gains, and this is reflected in the ratings of the Earlens system relative to their own hearing aids, as shown in Figures 12 and 13. However, comparison with the participants’ own hearing aids is difficult for several reasons. There was no control over what these hearing aids were, how well they were fitted, or how old they were. Nevertheless, all had been fitted by qualified audiologists or hearing aid dispensers and are representative of the range of air conduction devices that are worn in everyday life. Another consideration is the placebo effect, which can have a significant effect on subjective hearing aid comparisons (Bentler et al. 2003; Dawes et al. 2011, 2013). It was not possible to conduct a blind or double-blind comparison because the presence of the lens on the TM and the physical appearance of the processor and light tip differed significantly from those for the participants’ own hearing aids. Alternative techniques might be used in future studies, for example, comparing outcomes with the Earlens system programmed to have full versus limited bandwidth, which could be done double blinded.

The APHAB results suggested that problems with aversiveness were significantly less for Earlens than for the participants’ own hearing aids. This happened despite the fact that the former provided much more gain than the latter over the extended HFs. Evidently, the restoration of audibility for the extended HFs did not come at the cost of an overall increase in aversion.

Although the majority of the participants were previous hearing aid users, the final gains for the extended HF range were closer to the new-user settings than to the experienced-user settings from the CAM2 fitting algorithm. Sometimes, the HF gains were slightly reduced to avoid acoustic feedback, but the main reasons for the adjustments were participant reports of sharp sound quality, harsh onsets, sounds being too loud or uncomfortable, and distortion of /s/ and other HF sounds. These findings may indicate a need to modify the CAM2 fitting method to use somewhat reduced gains over the extended HF range. It may be the case that the gains recommended for new users are actually appropriate even for experienced users in that frequency region. Alternatively, these findings may reflect lack of familiarity with extended HF audibility as air conduction hearing aids, even if fitted to targets, usually do not provide audibility in that frequency region. Activation of acclimatization in the hearing aid programming may increase the likelihood that participants prefer the CAM2 targets for experienced users. In the current study, the subset of participants who had acclimatization activated was relatively small, and their fully-acclimatized settings at the 90-day visit were still ≈10 dB below the prescribed output at 8 to 10 kHz. A different acclimatization algorithm, for example, with a longer time course or smaller step size, may be more effective.

As described earlier, the initial fitting of the Earlens system involves a light-calibration procedure that is conceptually similar to real ear measurement for conventional air conduction hearing aids. However, unlike the latter, the light-calibration procedure for the Earlens system is obligatory; the fitting cannot proceed until the light-calibration has been completed. Thus, the person doing the fitting cannot save time by skipping the procedure. Furthermore, once the light-calibration procedure has been completed, the target gains can be implemented very accurately, probably more accurately than with conventional air conduction devices, for which the achieved gains often differ markedly from the target gains (Aazh & Moore 2007b; Aazh et al. 2012).

Although acoustic feedback can occur with the Earlens system, it is much less of a problem than for conventional air conduction hearing aids because sound radiation from the ear canal is small when the TM is driven directly (Puria 2003; Fay et al. 2006; Levy et al. 2013). The default program settings included activation of the feedback cancellation system for all programs except music, and for many participants, it was not necessary to activate the feedback cancellation system for the music program. This is advantageous as feedback cancellation systems often have deleterious effects on sound quality when listening to music (Freed & Soli 2006; Madsen & Moore 2014).

Individual differences in subjective ratings of the benefit of the Earlens system relative to unaided listening or to their own hearing aids as determined using the APHAB questionnaire (see Fig. 9) or in the comparison or satisfaction ratings determined using the Earlens Patient Satisfaction Questionnaire (Figs. 10–13) were mostly not correlated with any of the audiometric measures or with how close gain settings were to the CAM2 prescription. Thus, benefit from and preferences for the Earlens system were not lower for participants with severe hearing loss than for participants with mild hearing loss. It appears that the Earlens system can be of benefit to participants with a wide range of severity and patterns of hearing loss and for a range of gain settings.

A majority of the participants in the two studies had audiometric thresholds that did not exceed 65 dB HL for frequencies up to 4 kHz (see Fig. 2). It is unlikely that these participants had dead regions starting at 4 kHz or below (Aazh & Moore 2007a; Vinay & Moore 2007). However, a significant proportion of the participants had audiometric thresholds of 70 dB HL or more above 4 kHz, and it is likely that at least some of these had dead regions above 4 kHz. It remains unclear to what degree participants with dead regions starting at or above 4 kHz benefit from amplification over the extended HF range. It has been recommended that for a person with a continuous HF dead region, amplification should be provided for frequencies up to about 1.7 times the edge frequency of the dead region (Vickers et al. 2001; Baer et al. 2002). According to this guideline, for a person with a dead region starting at 4 kHz, amplification for frequencies up to about 7 kHz would be beneficial. However, this remains to be assessed. The Earlens system can provide the gains and output levels needed to restore audibility at HFs even for people with severe hearing loss, but further work is needed to establish the benefits of this, using participants who have been assessed using the TEN(SPL) test (Moore et al. 2000) or fast psychophysical tuning curves (Sek & Moore 2011) to determine whether or not they have a HF dead region, and, if so, what its edge frequency is.

The FGs measured at the start of each study corresponded reasonably well with the programmed IGs for soft speech for frequencies up to about 4 kHz, as should be the case if the system is calibrated accurately (see Figs. 6 and 7; compare the filled squares and the open squares with four points). For frequencies from 6 to 10 kHz, the FGs fell a little below the programmed IGs for soft speech. The reasons for this are not clear and may include calibration error, threshold measurement error, or output limiting due to the combination of high CTs and IGCT settings. The discrepancy may also reflect the fact that the effective microphone response at HFs depends strongly on the exact position of the photon processor relative to the pinna and on the anatomy of the individual’s head and pinna. For example, if the photon processor is positioned so that there is not a direct “line of sight” between the microphones and the loudspeaker used for the measurements of FG, as was the case for some of the participants in these studies, this will reduce the sound level at the microphone over the extended HFs, leading to a lower measured FG.

The FGs measured at the end of each study remained relatively high (Table 2). The decrease seen in study 1 was in part due to audiologist adjustment after First Fit Initial measurements. However, as can be seen more clearly in the data from study 2, despite a small increase in programmed settings, the mean FG decreased slightly. Again, the reasons for this are not clear. It is possible that the calibration changed by the end of the study due to some of the factors listed in the prior section, or that the microphone sensitivity decreased over time at HFs, due to the accumulation of debris on the diaphragms of the microphones or around the microphone ports. This may also occur with conventional air conduction hearing aids (Jensen 2004), but the effect would be less obvious because of their more limited frequency range.

Given the growing body of literature on the potential benefits of restoring the audibility of HF components in speech and music, from improved sound quality to improvements in speech understanding in complex environments, the results of these first studies of experience in the real-world with a hearing aid that can provide broadband amplification are promising. However, further research is needed to optimize HF fitting algorithms such as CAM2 and to explore the perceptual effects of modifying parameters, such as compression ratios, CTs, and time constants. The Earlens system currently incorporates slow-acting compression, which appears to be a “safe” option for most people with hearing impairment (Gatehouse et al. 2006a, 2006b) and which is preferred by many people with hearing impairment for listening to speech and by most people with hearing impairment for listening to music (Moore & Sek 2016b). However, it is possible that it would be beneficial to use fast-acting compression over the extended HFs, for which loudness recruitment tends to be most pronounced. This might reduce the incidence of complaints that some transient speech sounds (like /t/ and /k/) are overemphasized.

In the past, the lack of ability to assess the potential benefits of amplification of the extended HFs in real-world situations has been a major obstacle to research on this topic. The development and clinical trial of the Earlens system have allowed for a first glimpse into this area. The current two clinical trials have the limitation that they were not blinded, so the satisfaction measures may have been affected by placebo effects or biases. However, the demonstration of relatively large preferred gain values at HFs is an important first step. Future studies may take advantage of the capabilities of the Earlens system and perform double-blinded studies assessing the effects of amplification over the extended HF range.

## CONCLUSIONS

In summary, the results of the two studies show that the Earlens system can provide the gains and output levels prescribed by the CAM2 fitting method over the whole frequency range up to 10 kHz for participants with a wide range of hearing losses. The preferred gains over the extended HF range seem to be close to those prescribed by the CAM2 method for inexperienced users, even after extended use. These gains are substantial for frequencies above 5 kHz and are much higher than can be provided by the great majority of conventional hearing aids. Subjective results are promising although they should be treated with caution as the studies were not blinded. APHAB scores indicate that restoration of audibility at HF may not come at the expense of increased aversiveness. Questionnaire responses indicated that most participants were satisfied or very satisfied with the Earlens system, and most preferred the Earlens system to their own hearing aids.

## ACKNOWLEDGMENTS

The authors express their appreciation to the study participants, to the Clinical Investigators who treated and counseled the participants and collected the study data, and to Tim Streeter for assistance with fitting software data extraction and analysis. The research was partially supported by SBIR Phase IIB grant R44 DC 08499 from the National Institute on Deafness and Other Communication Disorders (NIDCD) of the National Institutes of Health. Portions of the data in this article were presented at the International Hearing Aid Research Conference (IHCON), Tahoe City, CA, August 10–14, 2016. Results from three Earlens Patient Satisfaction questions from study 1 were presented in a different format in Gantz et al. (2017). For study 2, functional gain, APHAB, and patient satisfaction results were presented for a different purpose or different format in McElveen et al. (Reference Note 1).

## Supplementary Material

## Footnotes

* All participants included in the current analysis had interaural threshold asymmetry less than the frequency-specific attenuation curve of the EAR foam earplugs used in both studies. Foam earplugs were inserted by the audiologist to ensure proper insertion for good attenuation values. One participant’s gain data in study 2 were excluded due to unilateral high-frequency loss such that the interaural asymmetry exceeded the earplug attenuation, and it appeared that unaided sound field thresholds were measured without masking.
