# Supplementary material for: Achieved Gain and Subjective Outcomes for a Wide-Bandwidth Contact Hearing Aid Fitted Using CAM2
Source: Ear Hear. 2019 Apr 26;40(3):741–56. doi: 10.1097/AUD.0000000000000661 (PMC6453763; doi:10.1097/AUD.0000000000000661)
Supplement: Supplementary file 1 [file aud-40-741-s001.docx]

**Relevant Excerpts of the Earlens Patient Satisfaction Questionnaires**

Responses to the following Earlens Patient Satisfaction questions were averaged to create the following summary subjective ratings for the correlation analysis: Average Satisfaction, Average Preference re Own Hearing Aid, Average Benefit re Unaided, and Average Benefit re Own Hearing Aid.

**Average Satisfaction (Study 1)**

6-point Likert scale: 1=Very Dissatisfied, 2=Dissatisfied, 3=Slightly Dissatisfied, 4=Slightly Satisfied, 5=Satisfied, 6=Very Satisfied

- How satisfied were you with the quality of sound delivered by your EarLens devices?
- How satisfied were you with the clarity of sound delivered by your EarLens devices?
- How satisfied were you with the naturalness of sounds delivered by your EarLens devices?
- How satisfied were you with the quality of music with your EarLens devices?
- How satisfied were you with your ability to understand speech in noisy environments with the EarLens devices?
- Overall, how satisfied were you with the performance of your EarLens devices in terms of addressing your hearing problems?

**Average Satisfaction (Study 2)**

5-point Likert scale: 1=Very Dissatisfied, 2=Dissatisfied, 3=Neutral, 4=Satisfied, 5=Very Satisfied

How satisfied were you with your EarLens devices regarding:

- Overall benefit for hearing
- Benefit for speech understanding
- Overall quality of sound
- Quality of speech sounds
- Quality of music

**Average Preference re Own Hearing Aid (Study 1)**

6-point Likert scale: 1=Very much prefer my other hearing aids, 2=Prefer my other hearing aids, 3=Slightly prefer my other hearing aids, 4=Slightly prefer Earlens, 5=Prefer Earlens, 6=Very much prefer Earlens

- Compared to your previous situation in which you wore your own hearing aids to treat your hearing impairment, do you prefer having the EarLens device?

**Average Preference re Own Hearing Aid (Study 2)**

5-point Likert scale: 1=Very much prefer my own hearing aids, 2=Prefer my own hearing aids, 3=Both about the same, 4=Prefer Earlens, 5=Very much prefer Earlens

- How would you rate the overall benefit and sound quality of EarLens compared to your own hearing aids?
- Compared to your own hearing aids, do you prefer having the EarLens device overall?

**Average Perceived Benefit re Unaided or Own Hearing Aid (Study 1)**

5-point Likert scale: 1=Worse, 2=No Change, 3=Slightly Improved, 4=Improved, 5=Very Much Improved

Rate the performance of your Earlens devices in terms of how well they improved your experience relative to your unaided condition in each of the following situations:

~OR~

Rate the performance of your Earlens devices in terms of how well they improved your experience relative to your own hearing aids in each of the following situations:

- Your hearing in noisy environments
- Your ability to participate in group conversations
- Your ability to tell where sounds are coming from
- Your experience listening to music
- Your experience listening to the TV
- Your experience listening to the radio
- Your effort required to carry on conversations
- Your overall quality of life

**Average Perceived Benefit re Unaided or Own Hearing Aid (Study 2):**

5-point Likert scale: 1=Earlens Much Worse, 2=Earlens Worse, 3=About the Same, 4=Earlens Better, 5=Earlens Much Better

Please rate the performance of EarLens compared to your experience with your own hearing aids in each of the following situations:

~OR~

If you had no hearing aid experience prior to the EarLens, please rate the performance of EarLens compared to unaided hearing in the following situations:

- Conversation in a noisy place or large group (e.g. loud restaurant)
- Smaller group conversations
- Listening to live music
- Listening to music on the radio/stereo at home
- Listening to the TV or talk radio at home
- Listening in an audience (movie, theater, place of worship)
- Listening to children
- Effort required to carry on conversations
- Overall quality of life
